# Detoxification of Ustiloxin A Through Oxidative Deamination and Decarboxylation by Endophytic Fungus *Petriella setifera*

**DOI:** 10.3390/toxins17020048

**Published:** 2025-01-22

**Authors:** Peng Li, Gan Gu, Xuwen Hou, Dan Xu, Jungui Dai, Yu Kuang, Mingan Wang, Daowan Lai, Ligang Zhou

**Affiliations:** 1 Department of Plant Pathology, College of Plant Protection, China Agricultural University, Beijing 100193, China; pengli1989@cau.edu.cn (P.L.); gugan@caas.cn (G.G.); xwhou@cau.edu.cn (X.H.); cauxudan@cau.edu.cn (D.X.); 2 State Key Laboratory of Bioactive Substance and Function of Natural Medicines, Institute of Materia Medica, Chinese Academy of Medical Science and Peking Union Medical College, Beijing 100050, China; jgdai@imm.ac.cn; 3 Department of Basic Veterinary Medicine, College of Veterinary Medicine, China Agricultural University, Beijing 100193, China; kuangyu@cau.edu.cn; 4 Innovation Center of Pesticide Research, Department of Applied Chemistry, College of Science, China Agricultural University, Beijing 100193, China; wangma@cau.edu.cn

**Keywords:** mycotoxin, ustiloxin A, cell-free extract (CFE), biotransformation, oxidative deamination, oxidative decarboxylation, *Villosiclava virens*, *Ustilaginoidea virens*, plant endophytic fungus, *Petriella setifera*, cytotoxic activity

## Abstract

Ustiloxins are a group of cyclopeptide mycotoxins produced by rice false smut pathogen *Villosiclava virens* (anamorph: *Ustilaginoidea virens*) which seriously threaten the safety production of rice and the health of humans and livestock. Ustiloxin A, accounting for 60% of the total ustiloxins, is the main toxic component. Biotransformation, a process of modifying the functional groups of compounds by means of regio- or stereo-specific reactions catalyzed by the enzymes produced by organisms, has been considered as an efficient way to detoxify mycotoxins. In this study, the endophytic fungus *Petriella setifera* Nitaf10 was found to be able to detoxify ustiloxin A through biotransformation. Two transformed products were obtained by using the cell-free extract (CFE) containing intracellular enzymes of *P. setifera* Nitaf10. They were structurally characterized as novel ustiloxin analogs named ustiloxins A1 (**1**) and A2 (**2**) by analysis of the 1D and 2D NMR and HRESIMS spectra as well as by comparison with known ustiloxins. The cytotoxic activity of ustiloxins A1 (**1**) and A2 (**2**) was much weaker than that of ustiloxin A. The biotransformation of ustiloxin A was found to proceed via oxidative deamination and decarboxylation and was possibly catalyzed by the intracellular amine oxidase and oxidative decarboxylase in the CFE. An appropriate bioconversion was achieved by incubating ustiloxin A with the CFE prepared in 0.5 mol/L phosphate buffer (pH 7.0) for 24 to 48 h. The optimum initial pH values for the bioconversion of ustiloxin A were 7–9. Among eight metal ions (Co^2+^, Cu^2+^, Fe^3+^, Zn^2+^, Ba^2+^, Ca^2+^, Mg^2+^ and Mn^2+^) tested at 5 mmol/L, Cu^2+^, Fe^3+^ and Zn^2+^ totally inhibited the conversion of ustiloxin A. In conclusion, detoxification of ustiloxin A through oxidative deamination and decarboxylation is an efficient strategy.

## 1. Introduction

Rice false smut (RFS) is a destructive fungal disease caused by the unique flower-infecting fungus *Villosiclava virens* (anamorph: *Ustilaginoidea virens*) [1]. The typical symptoms are visible after rice flowering with a big orange-to-green ball, commonly known as false smut ball (FSB) [2,3]. RFS can result in decreased production and severe economic loses in rice-growing areas around the world. In China, the annual average RFS incidence area was 3.06 million ha, resulting in a yield loss of 158.6 million kilograms per year, and the annual average area of prevention and control for RFS was 6.92 million ha from 2008 to 2016 [4]. Furthermore, the RFS pathogen can produce mycotoxins which are harmful to rice plants as well as to humans and animals. Therefore, RFS seriously threatens the high-yield and safe production of rice [4,5,6].

Three types of mycotoxins, including ustiloxins [7,8], ustilaginoidins [9,10,11] and sorbicillinoids [12,13], have been found in the RFS pathogen. Ustiloxins are water-soluble cyclopeptides [14], whereas both ustilaginoidins and sorbicillinoids are lipophilic polyketides [10,15]. Among them, ustiloxins have been considered to be the most toxic mycotoxins [5,14]. Six ustiloxins, including ustiloxins A, B, C, D, F, and G, have been isolated from water extract of rice FSBs and the cultured RFS pathogen *V. virens* [7,8,16]. Ustiloxin A accounted for 60% of the total ustiloxins [17].

Ustiloxins exhibit a variety of biological activities. The water extract (also called crude ustiloxin fraction) from rice FSBs was first reported to be toxic to rabbits by Suwa in 1915 [14]. The crude ustiloxins obtained from the water extract of FSBs and purified ustiloxin A were reported to cause liver and kidney damage in mice [18]. The water extract of rice FSBs showed mouse hepatotoxicity in vitro and in vivo via the apoptosis pathway by activating Nrf2/H_2_O-1 [19]. The virulence mechanism of ustiloxins was revealed to cause changes in CYP450 and DNA-replication-related genes in hepatocytes [20]. Ustiloxin A also inhibited the proliferation of renal tubular epithelial cells in vitro and induced renal injury by disrupting the structure and respiratory function of mitochondria in mice [21]. In addition, when zebrafish (*Danio rerio*) was exposed to ustiloxin A, growth was inhibited, and development was impaired. The aquatic toxicity of ustiloxin A to zebrafish was revealed to be obvious [22]. Ustiloxin A was detected to exist in human urine collected from the people living in the rice cultivation area of Enshi in China [23]. The quality of urinary ustiloxin A was significantly correlated with the activities of alanine aminotransferase in males. This male-biased hepatotoxicity of ustiloxin A was further confirmed in mice exposure experiment [24]. In addition, paddy water might be an important source for ustiloxin accumulation in rice seeds [25]. *Tetrahymena thermophila* is one of the fastest multiplying free-living eukaryotic cells. Exposure to 2.19, 19.01 and 187.13 mg/L of ustiloxin A in a culture medium for 5 days significantly reduced the theoretical population and cell size of *T. thermophila*. Furthermore, treatment with 187.13 mg/L of ustiloxin A changed the percentages of *T. thermophila* cells in different cell-cycle stages, and with an increased malformation rate compared with the control, suggesting the disruption of the cell cycle. The expressions of 30 genes involved in the enriched proteasome pathway were investigated, and seven cyclin genes and two histone genes were significantly down-regulated, which might be the modes of action responsible for the disruption of cell cycling due to exposure to ustiloxin A [26]. Ustiloxins also functioned as phytotoxins by inhibiting the radicle and plumule growth during the germination of rice, wheat, and maize seeds, even inducing an abnormal swelling of the seedling roots [8,27,28,29,30]. Based on the significant animal toxic, phytotoxic and cytotoxic activities of ustiloxins, it is urgent to remove or detoxify them from rice food and feed.

Some strategies such as physical, chemical and biological detoxification methods have been used to decrease or detoxify the toxicity of mycotoxins [31]. To the best of our knowledge, only phytocatalytic degradation of ustiloxin A was reported for detoxification by using wormlike graphitic carbon nitride [32]; there are no other reports about the detoxification of ustiloxin A. Mycotoxins are usually converted into less toxic or non-toxic products through biotransformation by either living organisms or their synthesized enzymes as biocatalysts [33,34,35]. In order to detoxify ustiloxins in rice food and feed, detoxification through biotransformation might be an efficient strategy.

**Figure 1:**
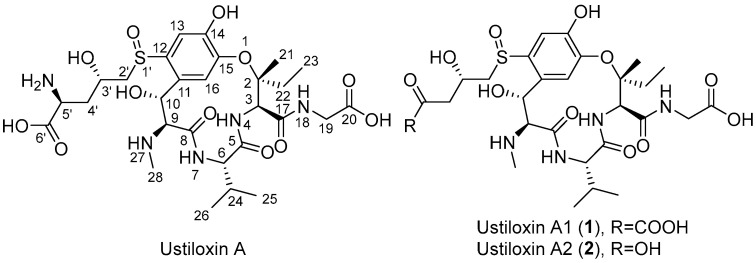
Structures of ustiloxins A, A1 (**1**) and A2 (**2**).

In the present study, the cell-free extract (CFE) of plant endophytic fungus *Petriella setifera* Nitaf10 was employed to convert ustiloxin A into two undescribed ustiloxin derivatives, namely, ustiloxins A1 (**1**) and A2 (**2**) (Figure 1). The cytotoxic activity of the transformed products was greatly decreased. The details of analysis, structural elucidation, and cytotoxic activities of two transformed compounds are reported here. The transformed mechanisms of ustiloxin A and the application of detoxified enzymes are also discussed.

## 2. Results

### 2.1. HPLC Analysis of the Biotransformed Products of Ustiloxin A

**Figure 2:**
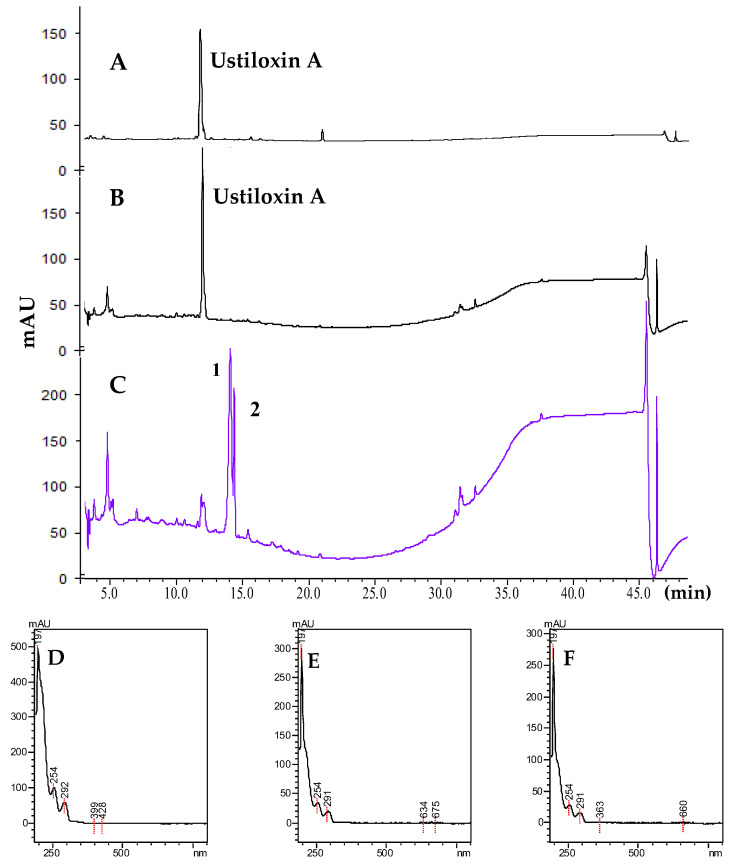
Incubation of ustiloxin A with the CFEs containing either extracellular of intracellular enzymes of *P. setifera* Nitaf10 for 36 h detected by HPLC analyses at 210 nm. (**A**) Ustiloxin A; (**B**) incubation of ustiloxin A with the CFE containing extracellular enzymes; (**C**) incubation of ustiloxin A with the CFE containing intracellular enzymes; (**D**) UV spectrum of ustiloxin A; (**E**) UV spectrum of **1**; (**F**) UV spectrum of **2**.

The cell-free extracts (CFEs) containing either extracellular or intracellular enzymes of *P. setifera* Nitaf10 were prepared and incubated with ustiloxin A for 36 h. The conversion progress was monitored by HPLC analysis (Figure 2). It was found that the extracellular enzymes of *P. setifera* failed to convert ustiloxin A (Figure 2B). However, two additional compounds (**1** and **2**), were detected in the CFE containing intracellular enzymes (Figure 2C). The UV spectra (Figure 2E,F) of these two compounds were identical to those of ustiloxin A (Figure 2D), indicating that these transformed products were analogs of ustiloxin A. Meanwhile, when ustiloxin A was incubated in the boiled CFE for 36 h, no product was detected. Hence, **1** and **2** should be the transformed products of ustiloxin A. Therefore, the CFE of *P. setifera* Nitaf10 containing intracellular crude enzymes was employed for ustiloxin A transformation in subsequent research.

### 2.2. Structural Identification of Transformed Products **1** and **2**

Compound **1**, which was isolated as a colorless amorphous powder, exhibited a prominent pseudomolecular ion peak at *m*/*z* 671.2288 [M–H]^−^ (calcd for C_28_H_39_N_4_O_13_S^−^, 671.2240) in the HRESIMS spectrum (Figure S1), indicating a molecular formula of C_28_H_40_N_4_O_13_S, with 11 degrees of unsaturation. The 1D and 2D NMR spectra of **1** are shown in Figures S2–S6. Analysis of its ^1^H NMR data (Table 1) and HSQC spectrum (Figure S4) revealed the presence of two aromatic protons (*δ*_H_ 7.60, 7.08), five heteroatomic substituted methine groups (*δ*_H_ 4.92, 4.82, 4.67, 4.30, 4.12), three methylene groups, five methyl groups (*δ*_H_ 2.78, 1.73, 1.08, 0.86, 0.77), and several aliphatic proton signals. The ^13^C NMR data (Table 1) displayed a total of 28 carbon resonances, which were assignable to six carbonyls (*δ*_C_ 190.3, 175.6, 170.6, 169.9, 169.0, 165.9), six sp^2^-hybridized carbons (*δ*_C_ 151.7, 145.5, 136.1, 127.9, 123.9, 113.6), one oxygenated quaternary carbon (*δ*_C_ 86.7), six methines including two oxygenated ones (*δ*_C_ 73.4, 62.0), three nitrogen-connecting ones (*δ*_C_ 66.0, 59.6, 59.1), one aliphatic methine (*δ*_C_ 28.4), and four methylenes (*δ*_C_ 63.5, 43.2, 38.7, 31.8), as well as five methyl groups (*δ*_C_ 31.6, 20.7, 17.8, 17.5, 7.3), with the aid of the HSQC spectrum (Figure S4). The above-mentioned results indicated that compound **1** had a bicyclic skeleton, which fulfilled the required unsaturation index.

**Figure 3:**
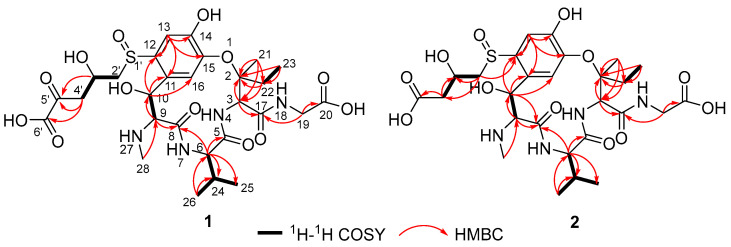
Key ^1^H−^1^H COSY and selected HMBC (H→C) correlations of ustiloxins A1 (**1**) and A2 (**2**).

Further analysis of the 2D NMR spectra of **1** (Figures S4–S6) showed that they were very similar to those of ustiloxin A [7,8]; the main difference was that the amino group at C-5′ of ustiloxin A was replaced by a ketone group in **1** (Figure 1). This was confirmed by the HMBC correlations from H-3′ to C-5′, and H_2_-4′ to C-5′ and C-6′ (Figure 3). Considering incubation of ustiloxin A in fungal cell-free extract (CFE), the absolute configuration of **1** was assumed to be the same as that of ustiloxin A. Thus, the structure of **1** was determined (Figure 1), and **1** was designated as ustiloxin A1.

Compound **2** was isolated as a colorless amorphous powder, with a molecular formula determined to be C_27_H_40_N_4_O_12_S (10 degrees of unsaturation), as determined by analysis of its HRESIMS spectrum (Figure S7). The 1D and 2D NMR spectra of **2** are shown in Figures S8–S11. Extensive analysis of 1D NMR data (Table 1) and 2D NMR spectra (Figures S10 and S11) revealed that **2** was very similar to compound **1**. The main difference was the absence of a ketone signal in the ^13^C NMR of **2**, which was in agreement with a unit with a mass of 28 less than that of **1**. This was confirmed by the observed HMBC correlation from H-4′ (*δ*_H_ 2.62) to C-5′ (Figure 3). Thus, the planar structure of compound **2** was elucidated, as shown in Figure 3. Considering incubation of ustiloxin A in the fungal cell-free extract solution, the absolute configuration of **2** was assumed to be the same as that of ustiloxin A. Thus, the structure of **2** was determined (Figure 1), and **2** was named ustiloxin A2.

### 2.3. Cytotoxic Activity of Ustiloxins A, A1 and A2

Ustiloxin A and its biotransformed products, ustiloxins A1 (**1**) and A2 (**2**), were assessed for their cytotoxic activity on five human cancer cell lines (Table 2). The median inhibitory concentration (IC_50_) value of ustiloxin A was smaller than that of ustiloxin A1 or A2, which indicated that the cytotoxic activity of the transformed products was weaker than that of the ustiloxin A. Among the three ustiloxins, ustiloxin A2 (**2**) showed the weakest cytotoxic activity.

### 2.4. Time Course of Ustiloxin A Bioconversion in the CFE of P. setifera Nitaf10

**Figure 4:**
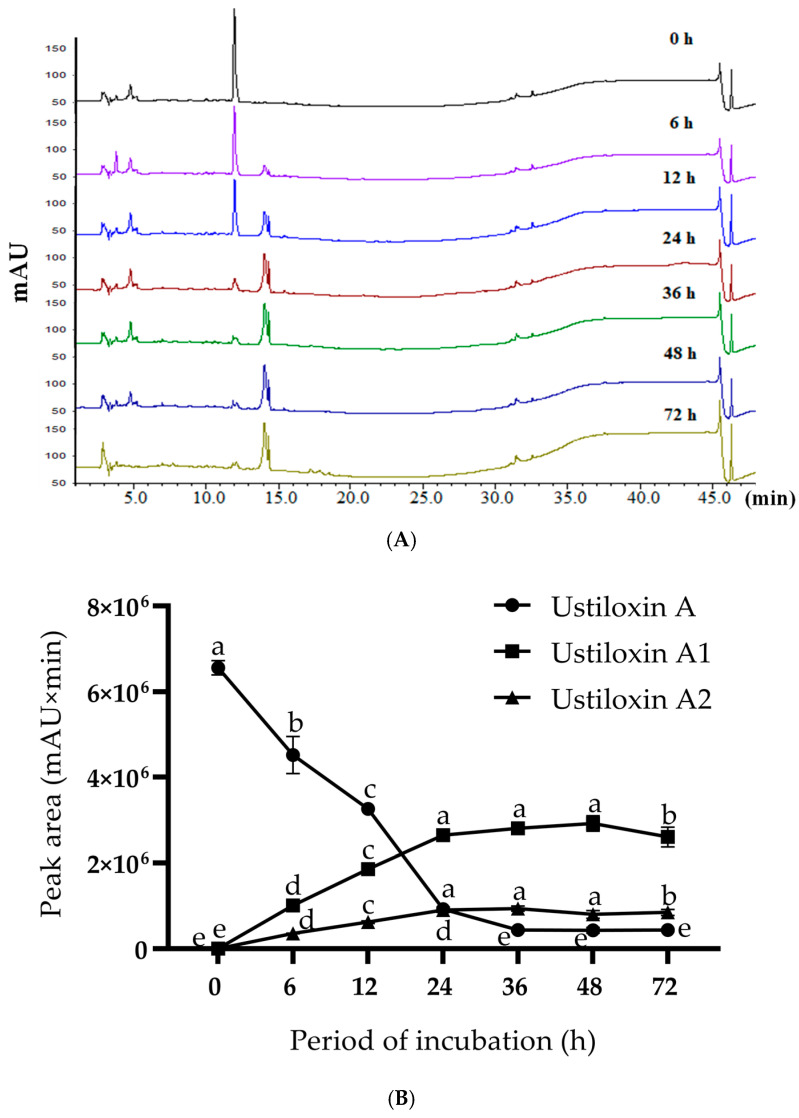
The bioconversion time course of ustiloxin A incubated in the CFE of *P. setifera* Nitaf10. (**A**) HPLC analyses of the samples collected after different periods of incubation and detected at 210 nm. (**B**) The relative quantities of ustiloxin A and its transformed products, ustiloxins A1 (**1**) and A2 (**2**), after different periods of incubation. The data in Figure 4B represent the mean ± standard deviation (n = 3). Different letters in each curve indicate that there are significant differences between the data when *p* ≤ 0.05.

The time course of ustiloxin A bioconversion in the CFE containing intracellular enzymes of *P. setifera* Nitaf10 is shown in Figure 4. The transformed products, ustiloxins A1 (**1**) and A2 (**2**), could be detected after 6 h of incubation. It was speculated that ustiloxin A was converted into ustiloxin A1 (**1**) through oxidative deamination catalyzed by an amine oxidase. Then, ustiloxin A1 (**1**) was further converted into ustiloxin A2 (**2**) through oxidative decarboxylation catalyzed by oxidative decarboxylase. The relative quantities of ustiloxins A1 (**1**) and A2 (**2**) reached their maximum values after 24 to 48 h of incubating ustiloxin A with the CFE prepared in 0.5 mol/L PBS (pH 7.0).

### 2.5. Effects of Initial pH Values on the Biotransformation of Ustiloxin A

**Figure 5:**
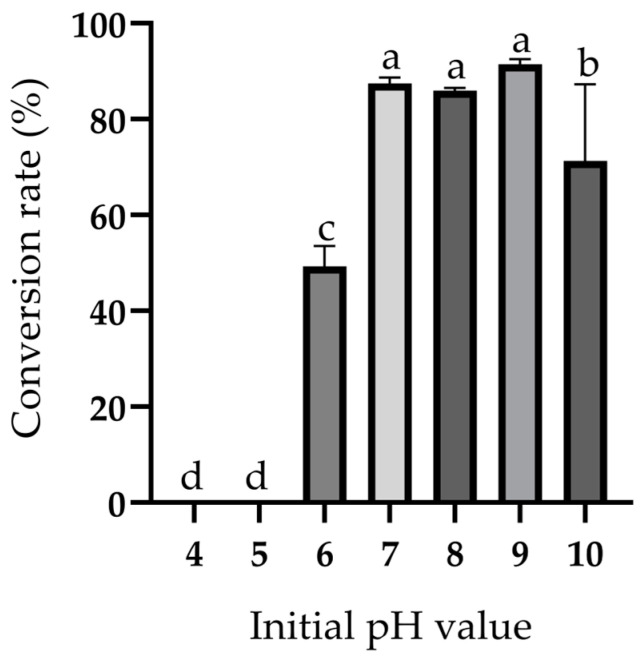
Effects of initial pH values on the biotransformation of ustiloxin A. The data in the figure represent the mean ± standard deviation (n = 3). Different letters indicate that there are significant differences between the data when *p* ≤ 0.05.

The effects of different initial pH values on the biotransformation of ustiloxin A were investigated with the conversion rates shown in Figure 5. The transformation did not happen at the initial pH values of 4 and 5. The conversion rate at pH 6 was 50%. At pH 7–9, the conversion rates were much higher. At pH 10, the conversion rate decreased to 70%. These results indicated that the pH of the CFE significantly affected biotransformation, and the optimum initial pH for ustiloxin A biotransformation was 7–9.

### 2.6. Effects of Metal Ions on the Biotransformation of Ustiloxin A in the CFE of P. setifera Nitaf10

**Figure 6:**
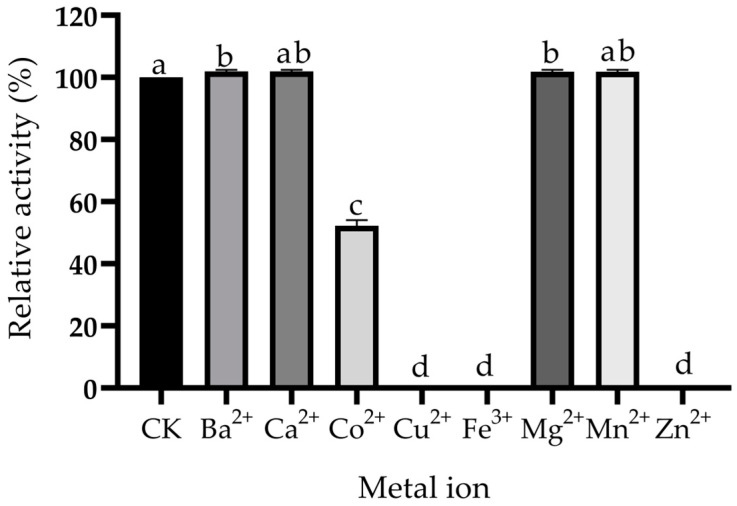
Effects of metal ions on the biotransformation of ustiloxin A. The concentration of each metal ion was at 5 mmol/L. The data in the figure represent the mean ± standard deviation (n = 3). Different letters indicate that there are significant differences between the data when *p* ≤ 0.05.

The effects of different metal ions, including Co^2+^, Cu^2+^, Fe^3+^, Zn^2+^, Ba^2+^, Ca^2+^, Mg^2+^ and Mn^2+^, at 5 mmol/L on the biotransformation of ustiloxin A in CFE containing intracellular enzymes of *P. setifera* Nitaf10 were investigated (Figure 6). The results indicated that Cu^2+^, Fe^3+^ and Zn^2+^ totally inhibited biotransformation, while the addition of Co^2+^ partially inhibited the transformation of ustiloxin A (ca. 50% inhibitory rate). Other metal ions, including Ba^2+^, Ca^2+^, Mg^2+^ and Mn^2+^, have a minor impact on the conversion reaction. The results in this study were similar to those of alaninol production through transformation using *Moraxella lacunata* WZ34 promoted by the ions of Ca^2+^, Mg^2+^ and Mn^2+^ [36].

## 3. Discussion

Mycotoxins can pose serious risks to animal and human health, such as carcinogenesis, teratogenicity, mutagenesis, and immunosuppression, and result in economic losses [37,38]. So, the detoxification of mycotoxins should be an efficient method. The detoxification of mycotoxins generally includes physical [39], chemical [40], and biological strategies [41,42,43]. Physical strategies, such as the application of adsorption agents, do not present a sufficient effect on mycotoxin detoxification [44]. Chemical strategies use bases, acids, oxidizing agents and aldehydes to modify the structures of mycotoxins, which has led to increased public concerns over the chemical residues in food and feed [45,46]. Although physical and chemical methods for mycotoxin detoxification have achieved varying degrees of success, the limited efficacy and losses of important nutrients still hamper their applications in food and feed industries [44,45,46]. In contrast, biological strategies, such as biotransformation and biosorption under mild and environmentally friendly conditions, have been recognized as promising solutions for the decontamination of mycotoxins [43,44].

The biotransformation of mycotoxins by living organisms (i.e., bacteria, fungi, plants and animals) and their isolated enzymes is a complex biochemical process due to their efficiency, specificity, and stereo selectivity to metabolize, destroy or deactivate mycotoxins into stable, less toxic or even nontoxic products [47,48]. According to the classes of chemical reactions, the main reaction types involved in mycotoxin biotransformation are as follows: (i) hydroxylation; (ii) oxido-reduction between alcohols and ketones; (iii) hydrogenation of the carbon–carbon double bond; (iv) de-epoxidation; (v) methylation; (vi) glycosylation and glucuronidation; (vii) esterification; (viii) hydrolysis; (ix) sulfation; (x) demethylation; (xi) deamination; and (xii) decarboxylation [34,35]. Biotransformation can be used not only for the detoxification of mycotoxins [33], but also for the production of novel compounds with biological activities [31,49].

Oxidative deamination of mycotoxins is the removal of an amino group from a molecule and its replacement with a hydroxyl or a ketone group catalyzed by amine oxidase, and it is also called transaminase or aminotransferase [50]. For example, hydrolyzed fumonisin B1 (HFB1) was converted into 2-keto HFB1 through oxidative deamination by the fungus *Exophiala spinifera* [51]. Fumonisin B4 (FB4) was transformed into fumonisins La4 (FLa4) and Py4 (FPy4) by amine oxidase from *Aspergillus* sp. [52]. Phytotoxic assay showed that both FLa4 and FPy4 were significantly less toxic in comparison to the fumonisin B4 by using a duckweed (*Lemna minor*) bioassay. The fumonisin amine oxidase of *Aspergillus niger* (AnFAO) was recombinantly expressed in *Escherachia coli*. AnFAO, which could convert fumonisin B2 (FB2) into low-toxicity fumonisin Py2 (FPy2), was considered a promising tool to remediate fumonisin-contaminated feed [53]. This demonstrated that *Aspergillus* fungi have the ability to produce oxidative deaminase that could be used for fumonisin detoxification [52,53]. The oxidative decarboxylation of mycotoxins is the removal of a carboxyl group in the form of carbon dioxide from a molecule and its replacement with a hydroxyl group catalyzed by oxidative decarboxylase. For example, vanillic acid was converted into 2-methoxybenzene-1,4-diol through oxidative decarboxylation by *Aspergillus flavus* [54]. Cyclohexylacetic aicd was converted into cyclohexanemethanol by the oxidative decarboxylase of *Candida maltose* [55].

The effects of pH on the bioconversion rates of exogenous compounds were obvious. The optimal pH values for the most enzyme-catalyzed reactions were between 4 and 9 [56]. The optimum initial pH values for ustiloxin A biotransformation were 7–9 in this study, which indicated that the enzyme-catalyzed transformation of ustiloxin A could tolerate a high pH (Figure 5). This is similar to that observed with the biotransformation of other compounds catalyzed by amine oxidases [57,58,59].

**Figure 7:**
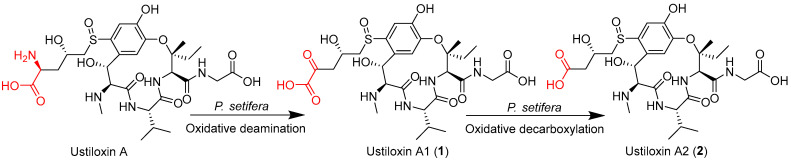
Biotransformation of ustiloxin A in the CFE of *P. setifera* Nitaf10.

In the present study, two transformed products, ustiloxins A1 (**1**) and A2 (**2**), of ustiloxin A were identified by using the CFE of plant endophytic fungus *P. setifera* Nitaf10. According to the time course of biotransformation shown in Figure 4, we speculated that ustiloxin A was firstly converted into ustiloxin A1 (**1**) by oxidative deamination under the catalysis of an amine oxidase produced by *P. setifera* Nitaf10. Then, ustiloxin A1 was gradually converted into ustiloxin A2 (**2**) by decarboxylation under catalysis of an oxidative decarboxylase. The biotransformation reaction of ustiloxin A in the CFE (pH 7.0) of *P. setifera* Nitaf10 is shown in Figure 7. The cytotoxic activities of the transformed products, ustiloxins A1 (**1**) and A2 (**2**), of ustiloxin A were greatly decreased. Ustiloxin A possessed a γ-hydroxy-δ-sulfinylnorvaline moiety at the aromatic ring (Figure 1), suggesting that the sulfinylnorvaline moiety provided significant binding interactions with tubulin [60]. So, the sulfinylnorvaline moiety has been considered the active group of ustiloxin cytotoxicity. The oxidative deamination and decarboxylation on ustiloxin A modified the structure of the sulfinylnorvaline moiety, and the cytotoxicity of the transformed products was greatly reduced. It was speculated that the amino group and carboxyl group in the sulfinylnorvaline moiety contributed to the cytotoxicity of ustiloxin A. The other toxic activities, such as phytotoxic and animal toxic activities, need to be further investigated.

Using the CFE of plant endophytic fungus *P. setifera* Nitaf10, it should be an efficient strategy to detoxify ustiloxin A through biotransformation. Both amine oxidase and oxidative decarboxylase produced by the endophytic fungus *P. setifera* Nitaf10 need to be purified to verify this conversion reaction. Furthermore, the recombinant and heterologous expression of the amine oxidase and oxidative decarboxylase from *P. setifera* Nitaf10 are also possible ways of accelerating the application of the biotransformation detoxification of ustiloxin A [61]. In addition to biotransformation using plant endophytic fungus, the management of ustiloxins by controlling their biosynthesis in fungi is another strategy, through methods such as the deletion of biosynthetic gene clusters and biosynthetic regulation for the production of ustiloxins [62].

## 4. Conclusions

In conclusion, the biotransformation of ustiloxin A was achieved by using a CFE containing intracellular enzymes of *P. setifera* Nitaf10, and two transformed products were structurally identified as novel ustiloxin analogs named ustiloxins A1 (**1**) and A2 (**2**) by spectroscopic analyses. The cytotoxic activity of the transformation products ustiloxins A1 (**1**) and A2 (**2**) was noticeably weaker than that of the precursor ustiloxin A, indicating they were detoxified products. The biotransformation of ustiloxin A was proposed to proceed via oxidative deamination and decarboxylation by the catalysis of an amine oxidase and an oxidative decarboxylase, respectively. An appropriate bioconversion was achieved by incubating ustiloxin A with the CFE prepared in 0.5 mol/L phosphate buffer (pH 7.0) for 24 to 48 h. The optimum initial pH values for the bioconversion of ustiloxin A were 7–9. Among eight metal ions (Co^2+^, Cu^2+^, Fe^3+^, Zn^2+^, Ba^2+^, Ca^2+^, Mg^2+^ and Mn^2+^) tested at 5 mmol/L, Cu^2+^, Fe^3+^ and Zn^2+^ totally inhibited the conversion of ustiloxin A. Detoxification of ustiloxin A through biotransformation should be an efficient strategy. This is beneficial for the safe production of rice food and feed by detoxifying ustiloxin A through biotransformation. However, the gene clone and heterologous expression of amine oxidase and oxidative decarboxylase, which were considered the biocatalysts from *P. setifera* Nitaf10, as well as enzymatic transformation and application in the detoxification of ustiloxins, need to be investigated in detail.

## 5. Materials and Methods

### 5.1. General Experimental Procedures

UV spectra were recorded on a TU-1810 UV−vis spectrophotometer (Beijing Persee General Instrument Co., Ltd., Beijing, China). High-resolution electrospray ionization mass spectrometry (HRESIMS) spectra were recorded on an LC 1260-Q-TOF/MS 6520 machine (Agilent Technologies, Santa Clara, CA, USA). ^1^H, ^13^C, and 2D NMR (HSQC, HMBC, ^1^H-^1^H COSY) spectra were measured on an Avance 500 NMR spectrometer (Bruker BioSpin, Zürich, Switzerland). Chemical shifts were expressed in δ (ppm) referring to the solvent residual peaks at *δ*_H_ 4.79, and coupling constants (*J*) were expressed in hertz (Hz). Column chromatography (CC) was performed on Sephadex G15 (40–70 μm; Amersham Pharmacia Biotech, Uppsala, Sweden). HPLC-PDA analysis was performed using a Shimadzu LC-20A instrument with an SPD-M20A photodiode array detector (Shimadzu Corp., Tokyo, Japan) and an analytic C_18_ column (250 mm × 4.6 mm i.d., 5 μm; Phenomenex Inc., Torrance, CA, USA). The HPLC elution conditions were as follows: 0–30 min, methanol from 10–100%, 30–40 min, 100% methanol-flushing column, 40–50 min, 10% methanol balance, flow rate was 1.0 mL/min, detection wavelength was 210 nm. Semi-preparative HPLC separation was carried out on a Lumtech instrument (Lumiere Tech. Ltd., Beijing, China) equipped with a K-501 pump (flow rate: 3 mL/min) and a K-2501 UV detector using a Luna-C_18_ column (250 mm × 10 mm i.d., 5 μm, Phenomenex Inc., Torrance, CA, USA).

All solvents were of HPLC grade (Fisher Scientific, Loughborough, UK). Ustiloxin A was prepared according to a previous report [8].

### 5.2. Endophytic Fungus Petriella setifera Nitaf10

The plant endophytic fungus *Petriella setifera* Nitaf10 (GenBank accession number KM095515) was isolated from the healthy roots of *Nicotiana tabacum* [63].

### 5.3. Preparation of Cell-Free Extract of P. setifera

Fungal cell-free extract (CFE) solutions include extracellular crude enzymatic solution and intracellular crude enzymatic solution. The activated fungus *P. setifera* Nitaf10 was cultured in PDB (potato dextrose broth) medium at 180 rpm for 3 to 5 days. The liquid fungal suspension was centrifuged at 12,000× *g* and 4 °C for 20 min. The supernatant containing extracellular crude enzymes was collected. Then, 10 mL of 0.5 mol/L pH 7.0 phosphate-buffered solution (PBS) was added to 10 g of the fresh precipitated mycelia, which was in an ice bath and subjected to ultrasonic treatment for 150 times (2 s oscillation and 2 s of intermittent for each time). Then, centrifugation was performed at 12,000× *g* and 4 °C for 20 min, and the supernatant, which was the CFE containing intracellular crude enzymes, was collected.

### 5.4. Incubation of Ustiloxin A in Cell-Free Extract of P. setifera

Briefly, 800 µL of CFE either containing extracellular crude enzymes or intracellular crude enzymes was added into the vial. Then, 200 µL of 500 µg/mL ustiloxin A was added; the control was the crude enzyme solution without ustiloxin A. This was react for 3 days at 28 °C, 180 rpm. After the reaction, the solution was filtered through a 0.22 µm microporous membrane for HPLC analysis, and compared with the control to see whether there was a different peak.

### 5.5. Isolation and Structural Identification of the Transformed Products of Ustiloxin A

After the biotransformation reaction was finished in 0.5 mol/L PBS (pH 7.0), the reaction solution was concentrated on Sephadex G-15 with ultra-pure water as the mobile phase to obtain crude transformed products, which were analyzed and compared by HPLC, and the pure compound was prepared by semi-preparative HPLC with 20% methanol water as the mobile phase. The detection wavelength was set at 210 nm. The purified compounds were structurally identified by analysis of UV, MS, ^1^H NMR, ^13^C NMR and 2D NMR data.

### 5.6. Cytotoxic Activity Assay

The cytotoxic activity of the compounds was tested against human carcinoma cells using the microculture methyl-thiazolyl-tetrazolium (MTT) assay as previously described [8]. The human carcinoma cell lines, including colon cancer cells (HCT-8), pancreatic cancer cells (PANC-1), gastric cancer cells (HGC-27), liver hepatocellular carcinoma cells (HepG2), and lung cancer cells (PC9), were tested on ustiloxins A, A1 (**1**) and A2 (**2**). Taxol was used as the positive control. All experiments were performed with three replicates.

### 5.7. Time Course of Biotransformation of Ustiloxin A by P. setifera Nitaf10

For the time course study of ustiloxin A biotransformation, 200 µL ustiloxin A solution (500 µg/mL) was added into 800 µL of 0.5 mol/L pH 7.0 PBS containing intracellular crude enzymes of *P. setifera* Nitaf10. The samples were harvested after 0 h, 6 h, 12 h, 24 h, 36 h, 48 h and 72 h of incubation at 28 °C and 180 rpm, and the reaction was terminated with an equal volume of methanol. After the solution was dried with nitrogen, 1 mL ultra-pure water was added to re-dissolve the residue. The solution was filtered through a 0.22 µm microporous membrane, and 10 µL of the solution was analyzed by HPLC with the conditions mentioned above. The relative quantity of each compound was represented by chromatographic peak area (mAU × min).

### 5.8. Effects of Initial pH Values on Biotransformation of Ustiloxin A by P. setifera Nitaf10

Furthermore, 5 mmol/L of buffer solutions with different pH values were prepared. They were pH 4 and 5 acetate buffer solutions, pH 6, 7 and 8 phosphate-buffered solutions, and pH 9 and 10 carbonate buffer solutions. Then, 200 µL of the ustiloxin A solution (500 µg/mL) was added into 800 µL of the buffer solutions with different pH values at 28 °C and 180 rpm for 3 days. The reaction was stopped with an equal volume of methanol. The subsequent steps were the same as the time course study of ustiloxin A biotransformation. The conversion rate of ustiloxin A was calculated as follows:

Conversion rate (%) = [(Quantity of ustiloxin A added—Quantity of ustiloxin A remained)/Quantity of ustiloxin A added] × 100(1)

### 5.9. Effects of Metal Ions on Biotransformation of Ustiloxin A by P. setifera Nitaf10

The metal ions in the form of CuCl_2_·2H_2_O, MgCl_2_·6H_2_O, CaCl_2_·2H_2_O, CoCl_2_·2H_2_O, MnCl_2_·4H_2_O, BaCl_2_·2H_2_O, ZnCl_2_ and FeCl_3_·6H_2_O were separately dissolved in 0.5 mol/L pH 7.0 PBS containing intracellular crude enzymes of *P. setifera* Nitaf10. Then, 200 µL ustiloxin A solution (500 µg/mL) was added into 800 µL of 0.5 mol/L pH 7.0 PBS containing intracellular crude enzymes and metal ions. The final concentration of each metal ion was maintained at 5 mmol/L. Sterile water of equal volume was used as the control, and the reaction lasted for 3 days at 28 °C and 180 rpm. The relative activity of each metal ion on the bioconversion of ustiloxin A was calculated as follows:

Relative activity (%) = (Conversion rate of ustiloxin A treated with a certain metal ion/Conversion rate of ustiloxin A untreated with metal ion) × 100(2)

### 5.10. Statistical Analysis

The data were expressed as mean values ± standard deviation (n = 3). The statistical analyses were performed using the SPSS statistical 19.0 software. One-way ANOVA with one-factor comparisons by LSD’s test was performed to test significant differences for comparing treatment means. Different letters indicated that the data were significantly different at *p* ≤ 0.05.

## Figures and Tables

**Table 1:** ^1^H NMR (500 MHz) and ^13^C NMR (125 MHz) data of ustiloxin A1 (**1**) and A2 (**2**) in D_2_O.

Position	1 (D_2_O)		2 (D_2_O)		Ustiloxin A (D_2_O) [7]	
	*δ*_C_, Type	*δ*_H_, Mult. (*J* in Hz)	*δ*_C,_ Type	*δ*_H_, Mult. (*J* in Hz)	*δ*_C,_ Type *^a^*	*δ*_H_, Mult. (*J* in Hz) *^a^*
2	86.7 C		86.4 C		87.2 C	
3	59.1 CH	4.82 m	58.7 CH	4.77 m	59.6 CH	4.83 s
5	170.6 C		170.6 C		171.0 C	
6	59.6 CH	4.12 d (10.0)	59.6 CH	4.05 d (10.2)	60.1 CH	4.13 d (10.0)
8	165.9 C		165.5 C		166.4 C	
9	66.0 CH	4.30 d (10.1)	66.0 CH	4.24 d (10.0)	66.7 CH	4.28 d (10.0)
10	73.4 CH	4.92 d (10.1)	73.4 CH	4.84 d (10.0)	73.9 CH	4.96 d (10.0)
11	127.9 C		127.6 C		128.0 C	
12	136.1 C		136.1 C		136.4 C	
13	113.6 CH	7.60 s	113.6 CH	7.54 s	114.0 CH	7.61 s
14	151.7 C		151.8 C		152.2 C	
15	145.5 C		145.4 C		146.0 C	
16	123.9 CH	7.08 s	124.2 C	7.01 s	124.2 CH	7.11 s
17	169.9 C		170.6 C		170.3 C	
19	43.2 CH_2_	3.78 s	41.1 CH	3.93 s	43.8 CH_2_	3.79 s
20	175.6 C		172.7 C		176.3 C	
21	20.7 CH_3_	1.73 s	20.8 CH_3_	1.68 s	21.1 CH_3_	1.77 s
22	31.8 CH_2_	2.22 dq (14.4, 7.2) 1.69 dq (14.4, 7.2)	31.8 CH_2_	2.16 dq (13.5, 6.5) 1.62 dq (13.5, 7.3)	32.1 CH_2_	2.24 dd (14.2, 7.2) 1.73 dd (14.2, 7.2)
23	7.3 CH_3_	1.08 t (7.2)	7.2 CH_3_	1.03 t (7.4)	7.8 CH_3_	1.09 dd (7.2, 7.2)
24	28.4 CH	1.87 m	28.4 CH	1.80 m	28.7 CH	1.92 dd (10.0, 7.0)
25	17.5 CH_3_	0.86 d (6.6)	17.5 CH_3_	0.80 d (6.6)	17.9 CH_3_	0.80 d (7.0)
26	17.8 CH_3_	0.77 d (6.6)	17.8 CH_3_	0.70 d (6.6)	18.3 CH_3_	0.88 d (7.0)
28	31.6 CH_3_	2.78 s	31.5 CH_3_	2.72 s	32.1 CH_3_	2.77 s
2′	63.5 CH_2_	3.34 m 3.02 m	63.3 CH_2_	3.30 dd (13.3, 8.7) 2.95 dd (13.3, 2.7)	64.8 CH_2_	3.33 dd (13.6, 10.0) 3.04 dd (13.6, 3.0)
3′	62.0 CH	4.67 m	62.7 CH	4.53 m	63.8 CH	4.39 m
4′	38.7 CH_2_	2.74 s	40.9 CH_2_	2.62 dd (13.3, 6.2)	36.7 CH_2_	2.22 ddd (15.0, 10.0, 8.0) 2.12 ddd (15.0, 8.0, 3.0)
5′	190.3 C		174.6 C		52.7 CH	4.01 dd (8.0, 8.0)
6′	169.0 C				174.4 C	

*^a^* The values of reference [7].

**Table 2:** Cytotoxic activity of ustiloxin A and its biotransformed products, ustiloxins A1 (**1**) and A2 (**2**), on human cancer cells.

Compound	IC_50_ (μmol/L)				
	HCT-8	PANC-1	HGC-27	HepG2	PC9
Ustiloxin A	2.81	3.59	3.62	11.94	1.85
Ustiloxin A1	5.95	6.93	4.59	20.05	15.01
Ustiloxin A2	6.73	13.04	15.80	21.91	21.80
Taxol (CK^+^)	4.18 × 10^−3^	1.53 × 10^−3^	1.76 × 10^−3^	4.50 × 10^−3^	2.51 × 10^−3^

Note: HCT-8, colon cancer cell line; PANC-1, pancreatic cancer cell line; HGC-27, gastric cancer cell line; HepG2, liver hepatocellular carcinoma cell line; PC9, lung cancer cell line; CK^+^ represents positive control.

## Data Availability

The original contributions presented in this study are included in this article/Supplementary Materials. Further inquiries can be directed to the corresponding author (L.Z.).

## Supplementary Materials

The following supporting information can be downloaded at: https://www.mdpi.com/article/10.3390/toxins17020048/s1, Figure S1: HRESIMS spectrum of ustiloxin A1; Figure S2: ^1^H NMR spectrum of ustiloxin A1; Figure S3: ^13^C NMR spectrum of ustiloxin A1; Figure S4: HSQC spectrum of ustiloxin A1; Figure S5: ^1^H-^1^H COSY spectrum of ustiloxin A1; Figure S6: HMBC spectrum of ustiloxin A1; Figure S7: HRESIMS spectrum of ustiloxin A2; Figure S8: ^1^H NMR spectrum of ustiloxin A2; Figure S9: ^13^C NMR spectrum of ustiloxin A2; Figure S10: HSQC spectrum of ustiloxin A2; Figure S11: HMBC spectrum of ustiloxin A2.
