# Supplementary material for: Detoxification of Ustiloxin A Through Oxidative Deamination and Decarboxylation by Endophytic Fungus Petriella setifera
Source: Toxins (Basel). 2025 Jan 22;17(2):48. doi: 10.3390/toxins17020048 (PMC11861864; doi:10.3390/toxins17020048)
Supplement: Supplementary file 1 [file toxins-17-00048-s001.zip › toxins-3398492-supplementary.pdf]

Supplementary Materials:

Detoxification of Ustiloxin A Through Oxidative Deamination and Decarboxylation by Endophytic Fungus *Petriella setifera*

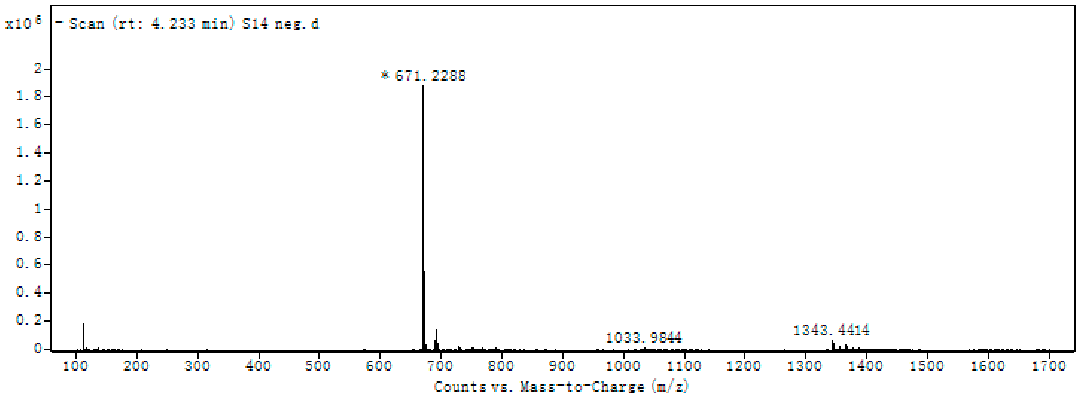

Figure S1. HRESIMS spectrum of ustiloxin A1 (**1**).

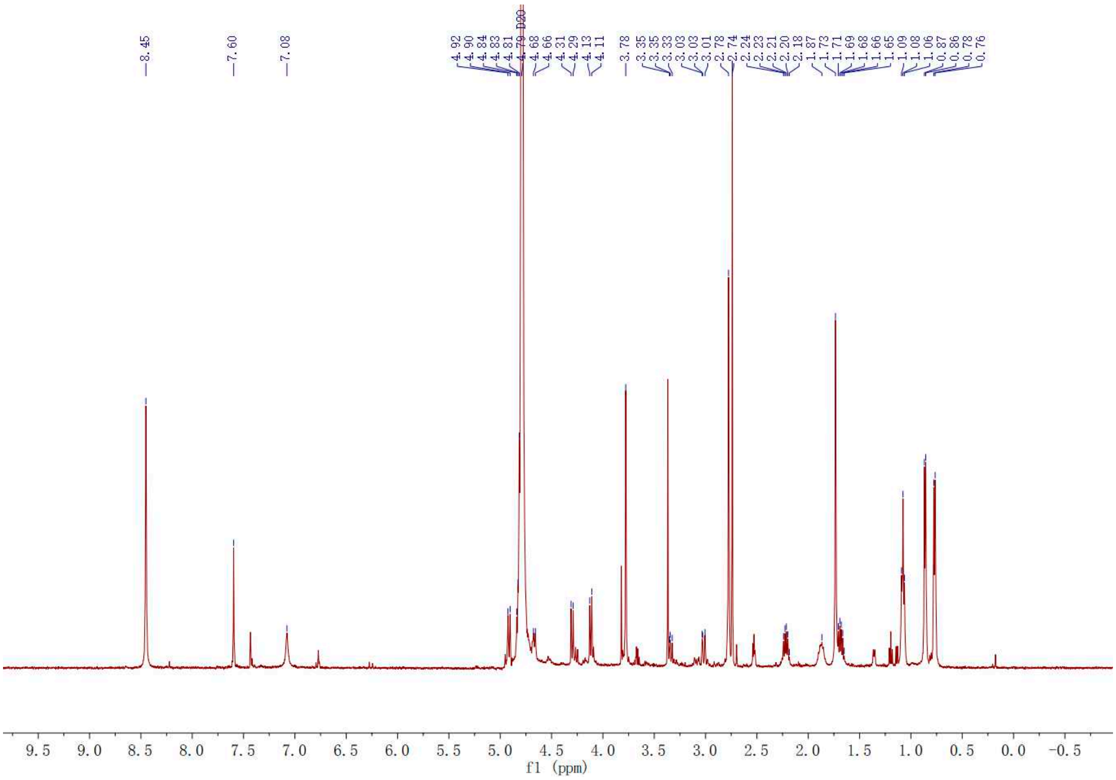

Figure S2. ^{1}H NMR spectrum of ustiloxin A1 (**1**) (D_2O, 500 MHz).

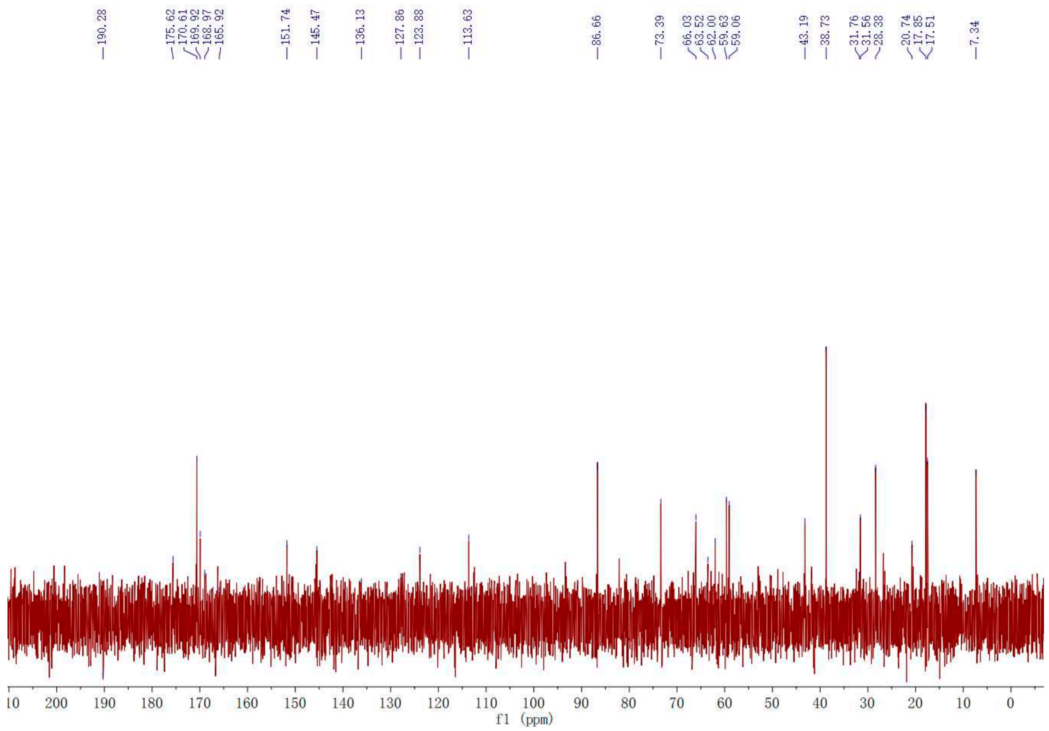

Figure S3. ^{13}C NMR spectrum of ustiloxin A1 (**1**) (D_2O, 125 MHz).

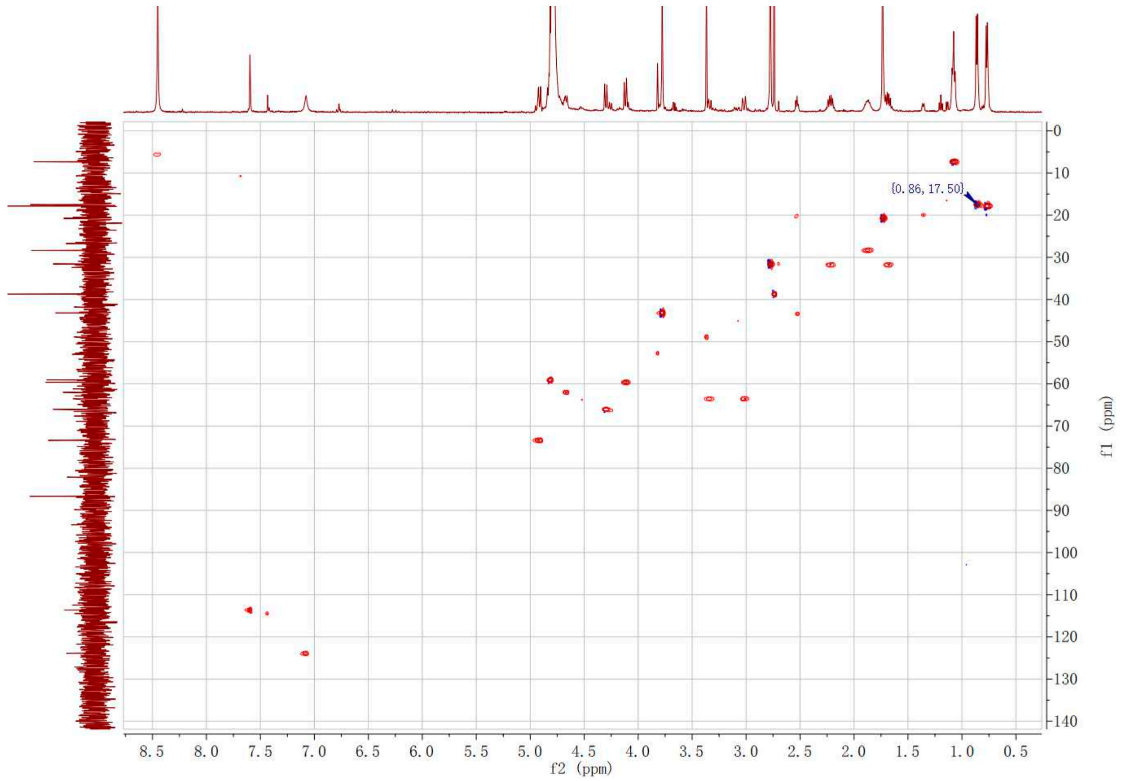

Figure S4. HSQC spectrum of ustiloxin A1 (**1**)

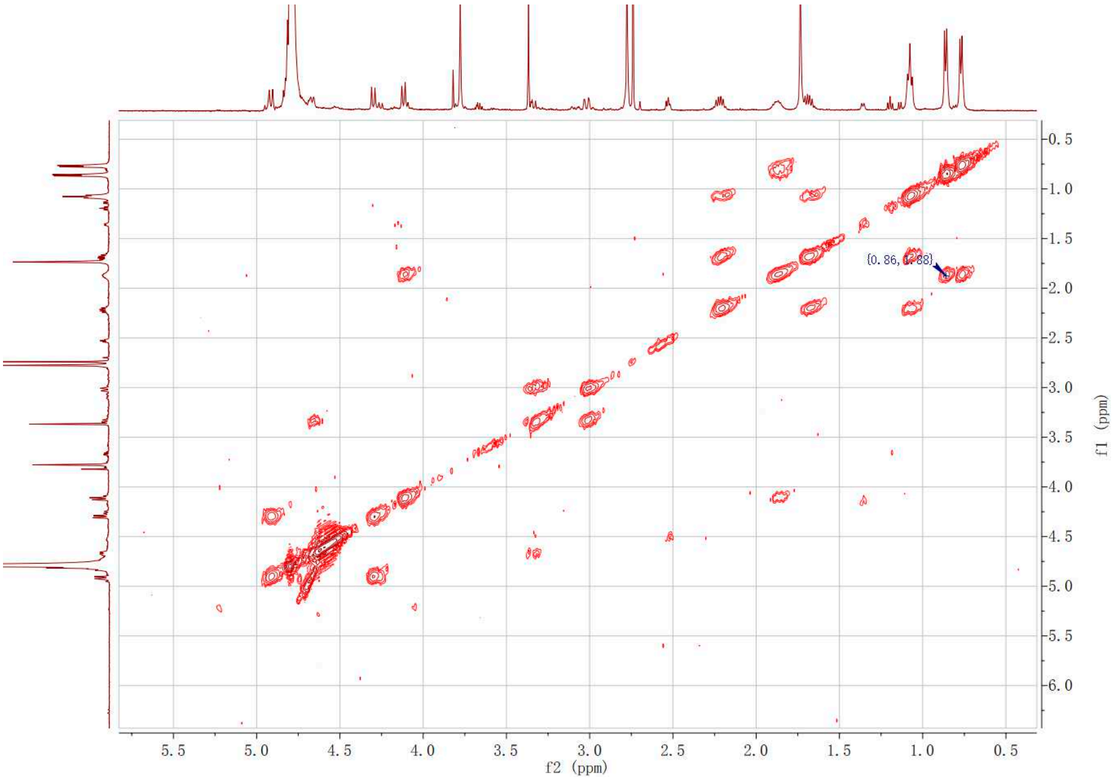

Figure S5. ^{1}H-^{1}H COSY spectrum of ustiloxin A1 (**1**).

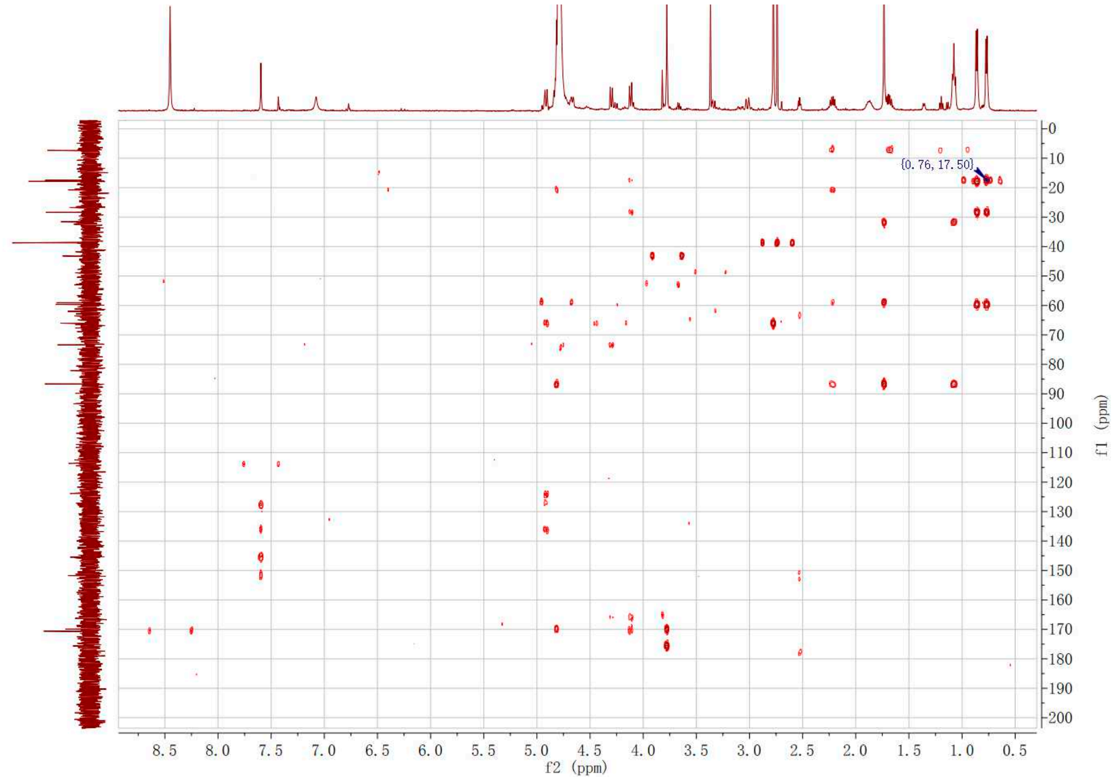

Figure S6. HMBC spectrum of ustiloxin A1 (**1**).

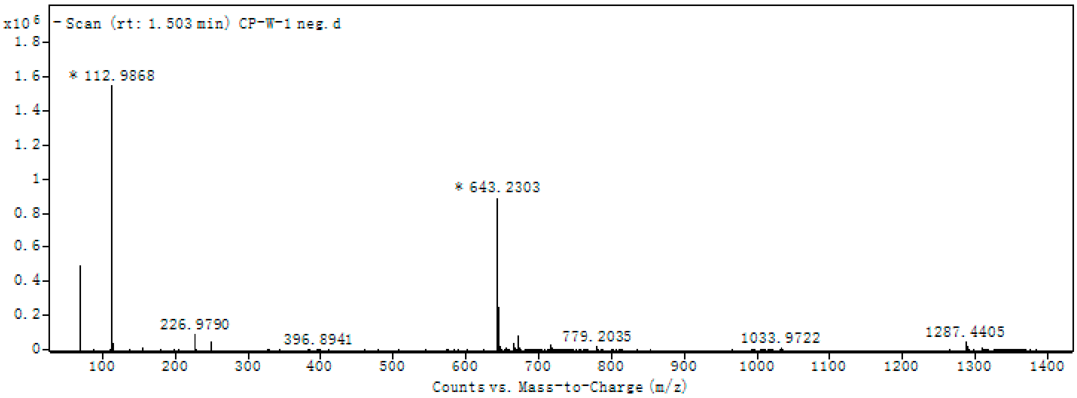

Figure S7. HRESIMS spectrum of ustiloxin A2 (**2**).

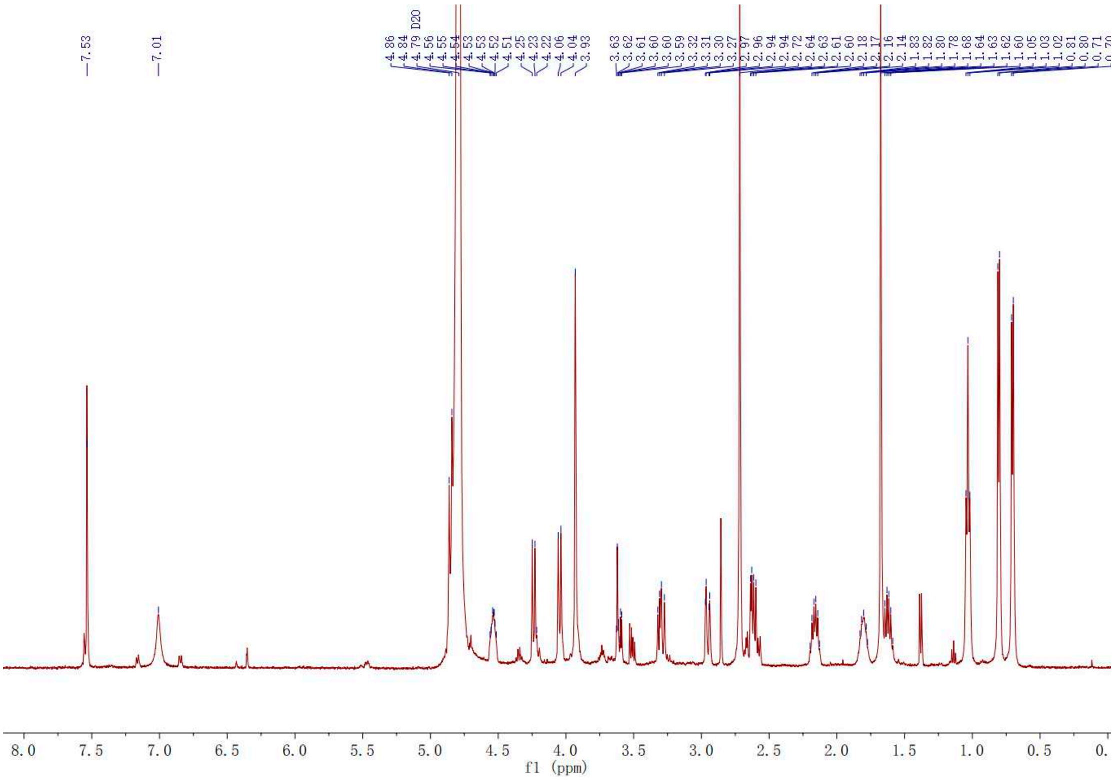

Figure S8. ¹H NMR spectrum of ustiloxin A2 (**2**) (D_2O, 500 MHz).

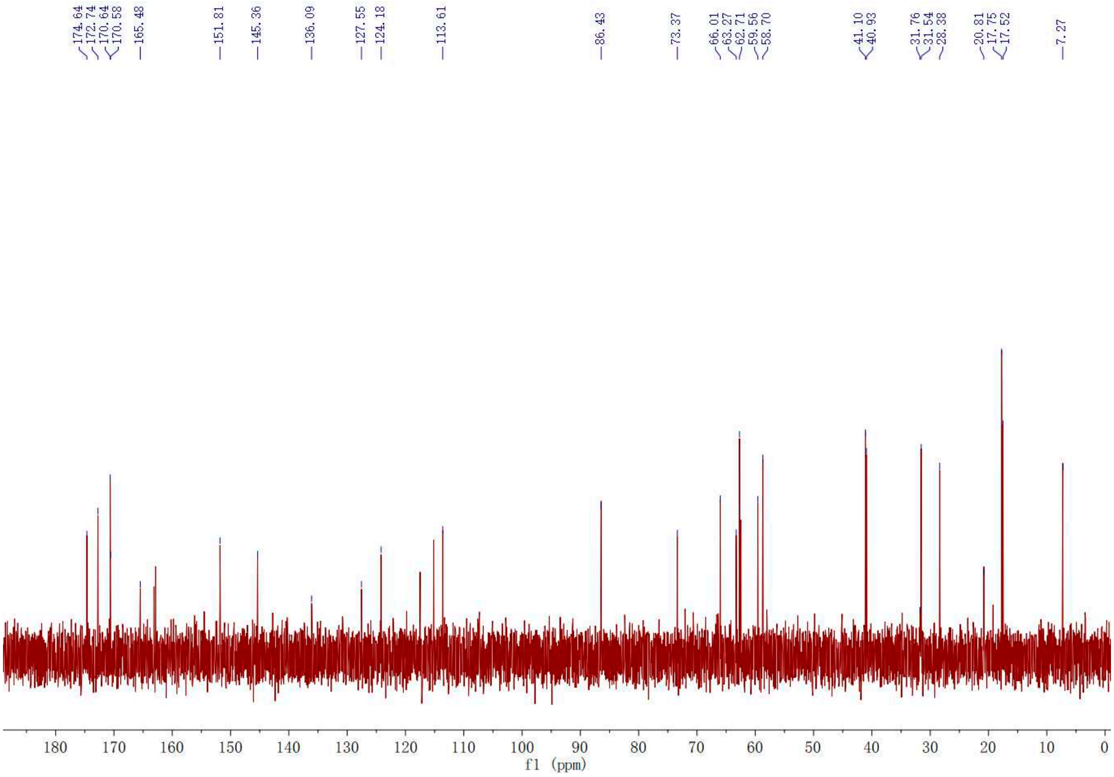

Figure S9. ^{13}C NMR spectrum of ustiloxin A2 (**2**) (D_2O, 125 MHz).

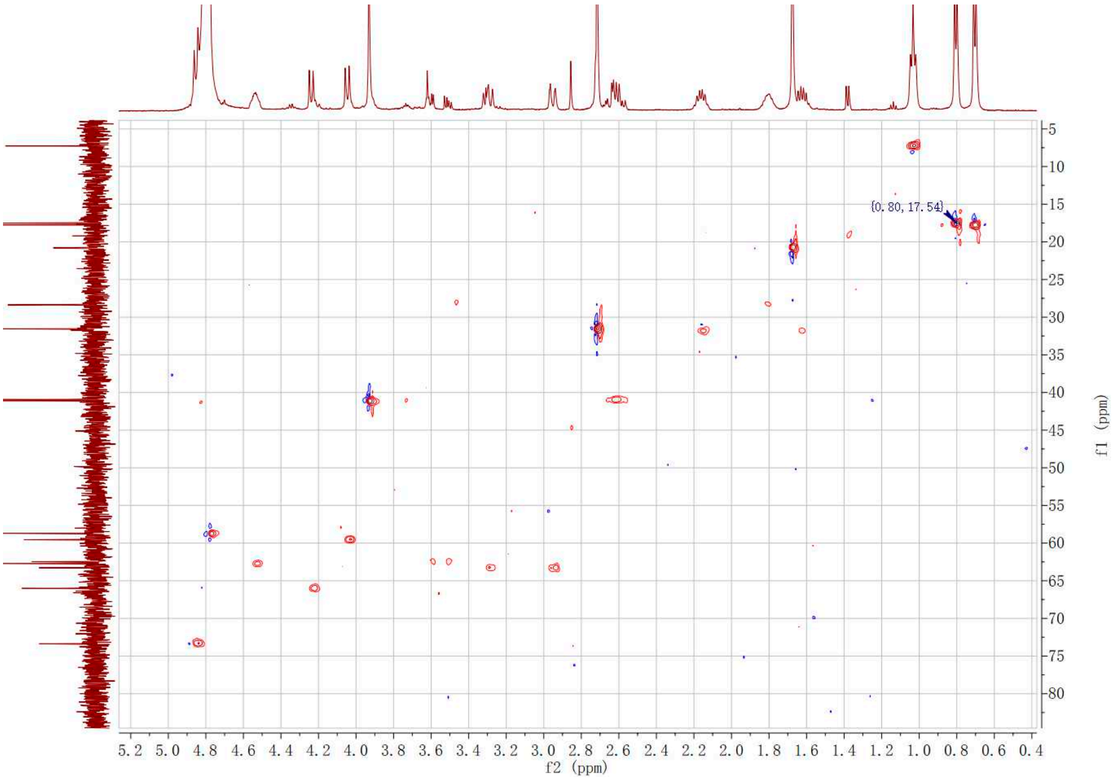

Figure S10. HSQC spectrum of ustiloxin A2 (**2**).

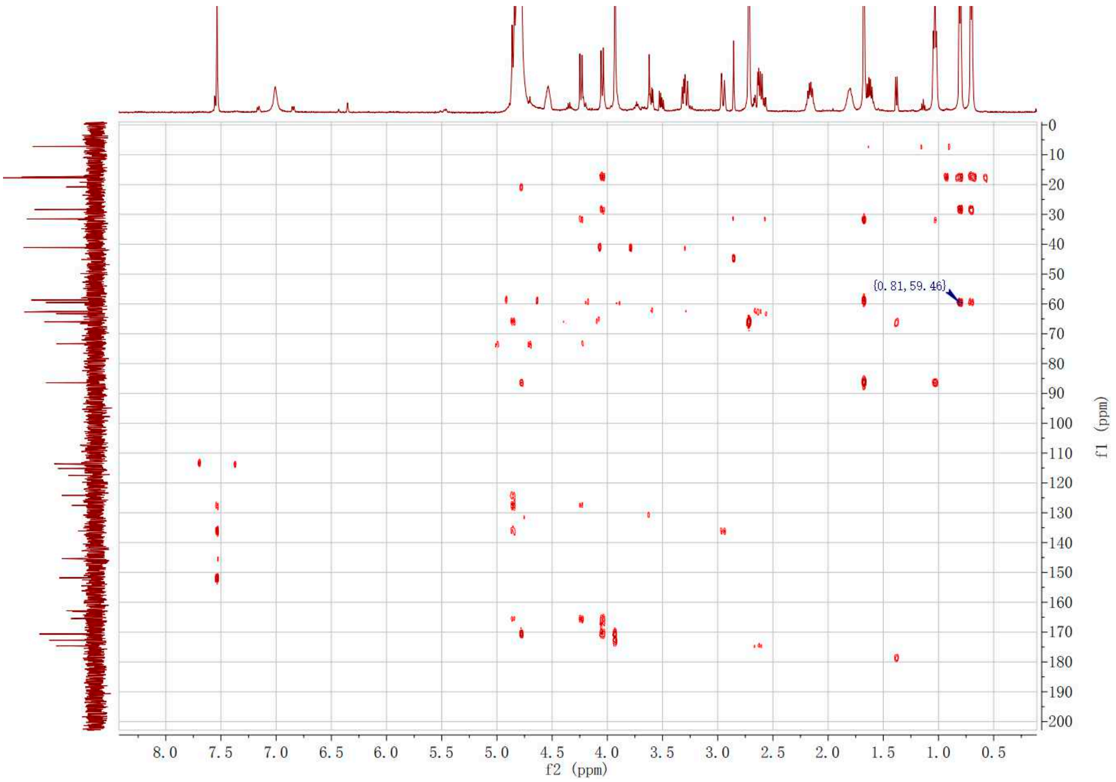

Figure S11. HMBC spectrum of ustiloxin A2 (**2**).
